# Floating Forearm Injury: Monteggia Variant Fracture With an Ipsilateral Distal Radius and Ulna Fracture

**DOI:** 10.7759/cureus.33263

**Published:** 2023-01-02

**Authors:** Zehong Chen, Sreenadh Gella, Kanthan Theivendran

**Affiliations:** 1 Trauma and Orthopaedics, Sandwell and West Birmingham Hospitals NHS Trust, Birmingham, GBR

**Keywords:** monteggia's fracture, floating forearm, surgical case report, distal radial and ulnar fracture, intraarticular fracture distal radius

## Abstract

Monteggia variant fracture is a Monteggia fracture (proximal third ulna fracture with radial head dislocation) with an associated radial head fracture, coronoid fracture or complex pattern of injury. We report a rare case of an 80-year-old lady with a right Monteggia variant fracture with an ipsilateral distal radius and ulna fracture leading to a floating forearm injury. To our knowledge, this is the first case report to describe this injury pattern. We describe the multidisciplinary team approach and detailed surgical technique in managing this rare and complex injury.

## Introduction

A Monteggia fracture is a fracture of the proximal third of the ulna with an associated radial head dislocation. They are rare and occur in 1-2% of all forearm fractures [1,2]. Monteggia variant fracture is a Monteggia fracture with an associated radial head, coronoid fracture, or complex pattern of injury [3]. Simultaneous fracture-dislocation of the ipsilateral elbow and wrist has rarely been described, especially in adults. Here, we report a rare and complex case of a floating forearm injury involving a Monteggia variant fracture with an ipsilateral distal radius and ulna fracture in an adult patient.

## Case presentation

An 80-year-old right-hand dominant lady was admitted to the emergency department following an unwitnessed fall while walking home. She was conscious and oriented but could not remember the exact mechanism of her fall. She has an extensive past medical history including osteoporosis, rheumatoid arthritis, and systemic lupus erythematosus (SLE). She had previously sustained a fracture to her right wrist and was treated conservatively for this. She is a non-smoker, mobilizes without any walking aids, and is independent in all activities of daily living. Apart from pain and swelling in her right wrist and elbow, she had no other injuries. These were closed injuries with no associated nerve or vessel injuries.

**Figure 1:**
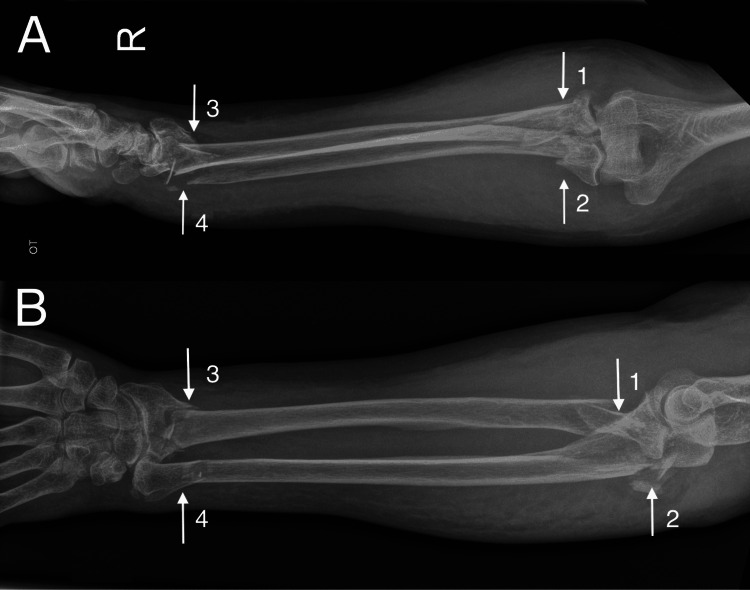
A: lateral radiograph of right forearm; B: anteroposterior radiograph of right forearm Arrow 1: Radial head fracture; Arrow 2: Olecranon fracture; Arrow 3: Distal radius fracture; Arrow 4: Distal ulna fracture

She underwent radiographs of her right forearm that showed displaced and comminuted intra-articular fractures of the distal radius, distal ulna shaft fracture, proximal ulna fracture, radial head fracture dislocation, and radial neck fracture extending into the shaft of the radius (Figures 1A, 1B). She was diagnosed with a Monteggia variant injury with an ipsilateral fracture of the distal radius and ulna. Her pattern of fractures sustained meant that there was discontinuity between her wrist and elbow joints leading to a floating forearm injury.

**Figure 2:**
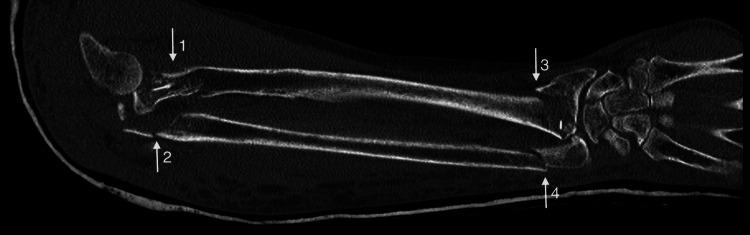
Coronal CT of right forearm Arrow 1: Radial head fracture; Arrow 2: Olecranon fracture; Arrow 3: Distal radius fracture; Arrow 4: Distal ulna fracture

Plain radiographs were supplemented with CT scans of her right forearm including the right wrist and elbow to aid surgical planning (Figure 2). 

The case was discussed in a multidisciplinary meeting consisting of several upper-limb orthopedic specialists, physiotherapists, and the medical team. The benefits and risks of operative and conservative management were discussed with the patient. It was eventually decided that the plan would be to fix the distal radius fracture first with a locking plate, assess the stability of the distal ulna fracture, and then fix the proximal ulna fracture to correct the length and alignment of the ulna, thereby guiding the correct height of the radial head with a long-stem radial head replacement. Given the complex nature of the injury, dual-consultant operating was advised to improve outcomes and reduce surgical time. It was felt this approach would give her the best functional outcome and pain relief with early rehabilitation.

**Figure 3:**
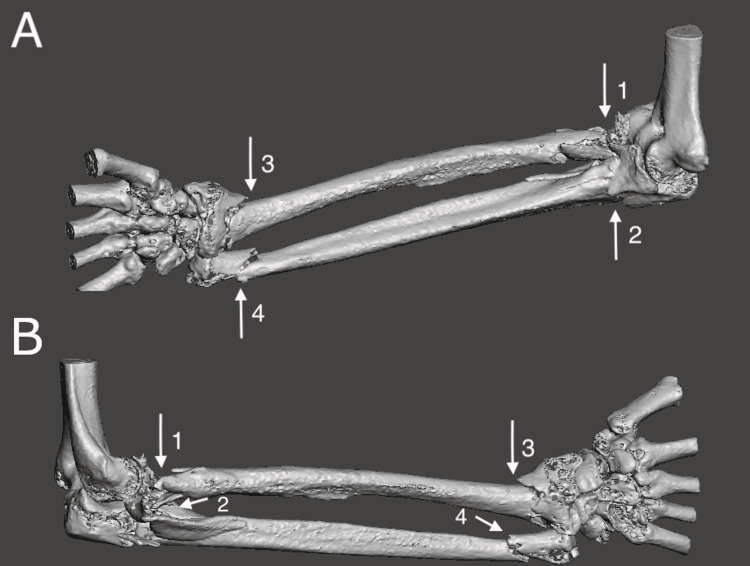
A: Medial view of the 3D CT reconstruction of the right forearm; B: Lateral view of the 3D CT reconstruction of the right forearm Arrow 1: Radial head fracture; Arrow 2: Olecranon fracture; Arrow 3: Distal radius fracture; Arrow 4: Distal ulna fracture

Meticulous 3D preoperative planning with MERGE PACS™ software (IBM, Watson Health, USA) allowed visualization of where the plate and screws should be applied and how the radial head would be replaced with a long stem implant (Figures 3A, 3B).

The patient was placed supine on the operating table with the arm placed on an arm board. A standard preoperative WHO checklist was performed before the start of the surgery. Intravenous tranexamic acid, flucloxacillin, and gentamicin were given at induction of anesthetic before skin incision. Pre-wash was performed with aqueous chlorhexidine and subsequent double skin preparation with 2% alcoholic chlorhexidine and a sterile drape was applied to the right arm.

**Figure 4:**
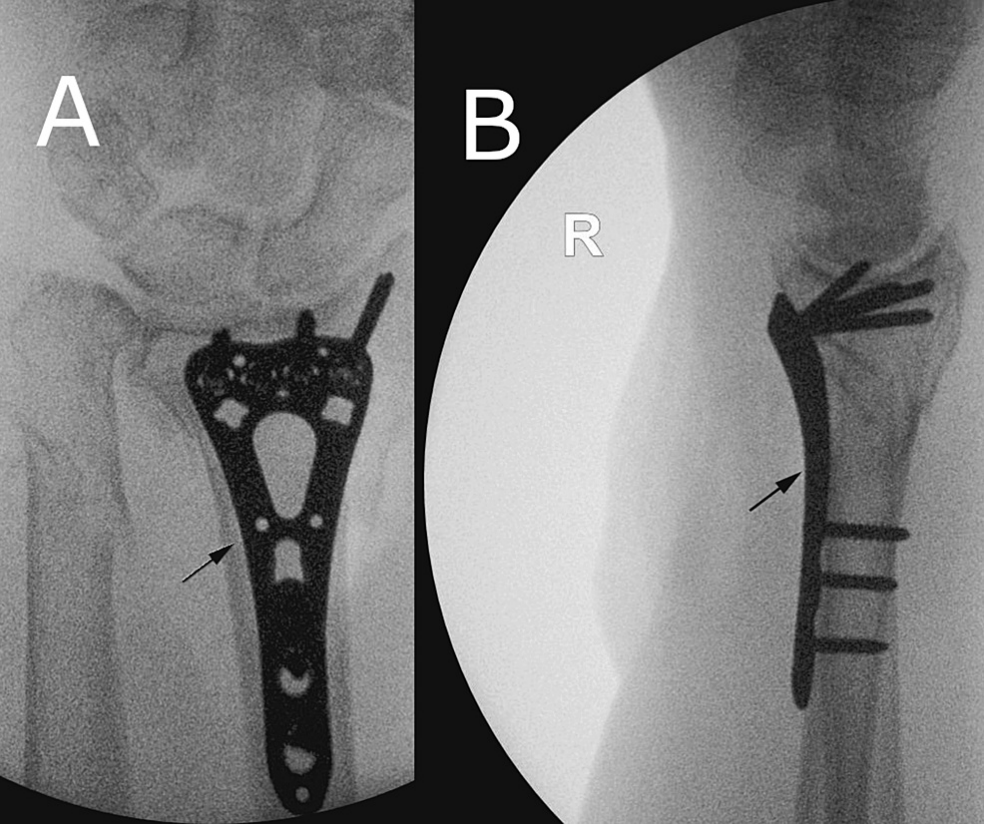
A: Anteroposterior intra-operative image intensifier (II) views of the wrist; B: Lateral intra-operative II views of the wrist Distal radius plate illustrated with arrows

The wrist fracture was addressed first with the flexor carpi radialis approach to the distal radius. The median nerve and radial artery were protected throughout the procedure. The pronator quadratus muscle was elevated off the distal radius to expose the fracture fragments. The fracture fragments were cleaned and reduced using the elevator and hitched onto the volar surface of the distal radius. A distal radius volar locking plate (DePuy Synthes, Raynham, USA) was applied on the volar side and held with K-wires through the plate. This was checked on image intensifier (II) with good reduction and position achieved. Distal locking screws were inserted into the distal fragment whilst reducing the distal fragment onto the volar plate. One cortical screw and two locking screws were inserted into the shaft of the radius. K-wires were then removed. This was then checked on II and confirmed excellent reduction, fixation and stability (Figures 4A, 4B). The distal ulna fracture was undisplaced and stable on examination after distal radius fixation and, therefore, did not require fixation. 

**Figure 5:**
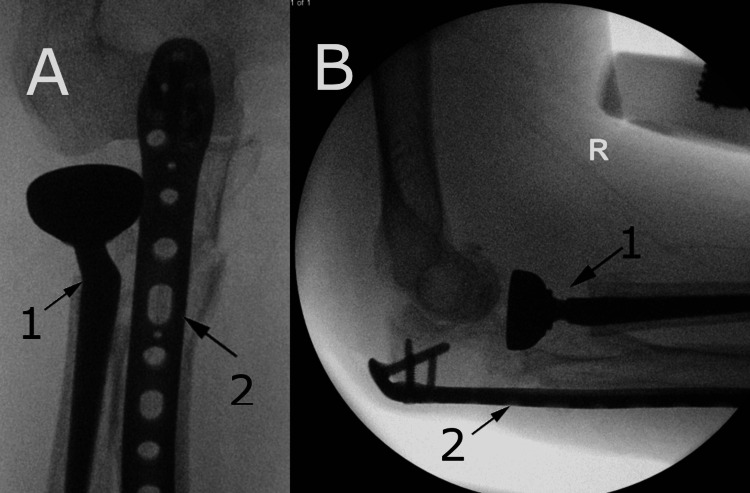
A: Anteroposterior intra-operative image intensifier (II) views of the elbow; B: Lateral intra-operative II views of the elbow Arrow 1: Radial head implant; Arrow 2: Pre-contoured olecranon plate

For the complex elbow fracture, a posterior midline skin approach was used avoiding the tip of the olecranon and medial and lateral full thickness skin flaps were created. The multifragmented olecranon fracture fragments were reduced and fixed with Fibrewire (Arthrex GmBH, Germany) cerclage sutures. An 11-hole, pre-contoured, right olecranon locking plate (Acumed, Portland, USA) was applied and fixed with non-locking and locking screws under II guidance. Bone graft was used in the olecranon bone defect from the remainder of bone from the excised radial head and secured with Surgicel (Johnson & Johnson Medical NV, Belgium). A window utilizing the Boyd approach through the anconeus and reflecting the supinator from its posterolateral attachment was performed to access the radial head and neck fracture. The posterior interosseous nerve was protected during this approach. The radial head was found to be multifragmented and not fixable. The fracture also extended to the shaft of the radius. Fibrewire cerclage sutures were placed around proximal radius fragments to hold and fix the radial shaft fracture fragments. The radial head remnant was then resected to allow for an 8 mm x 50 mm long-stem Acumed radial head replacement implant (Portland, USA). The radial canal was prepared with rasp and reamers. A definitive right radial head implant (Acumed, Portland, USA) with an 8 mm diameter x 50 mm long stem and a 24 mm radial head was inserted with cement in the correct orientation by aligning the laser line of the head of the definitive implant with the Lister's tubercle of the distal radius. Cement was used as the stem size was between the available stem diameters so cementation was chosen to provide secure primary fixation (Figures 5A, 5B). The fascia and skin were closed with sutures in layers and the elbow was placed in an above-elbow plaster of paris cast to let the wound heal and to aid soft tissue swelling management. 

**Figure 6:**
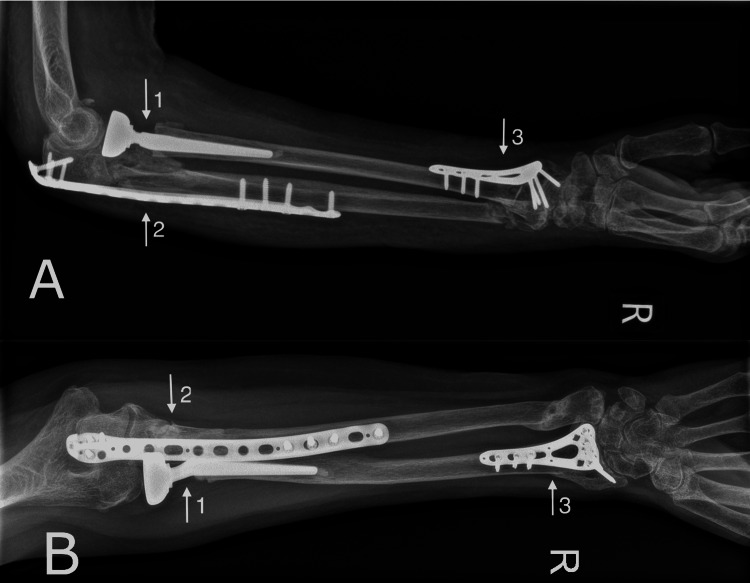
A: Lateral radiograph taken six months post surgery; B: Anteroposterior radiograph taken six months post surgery Arrow 1: Radial head replacement; Arrow 2: Pre-contoured olecranon plate; Arrow 3: Distal radius plate

The patient was reviewed two weeks post-surgery where sutures were removed and gentle range of movement physiotherapy exercises commenced. She was subsequently reviewed at six weeks, three months and her range of movement and QuickDASH score was assessed at six months post surgery. Her elbow range of movement in flexion from 0-150 degrees, range of supination from 0-85 degrees and range of pronation from 0-75 degrees. She had recovered most of her range of movement and her QuickDASH score was 36.4. Her Visual Analogue Scale (VAS) score for subjective pain was zero at rest and one while performing activities. Radiographs were also taken, which showed a healed fracture of the proximal ulna, distal radius, distal ulna, and a well-fixed radial head implant (Figures 6A, 6B). 

## Discussion

To our knowledge, this is the first case report to describe a Monteggia variant fracture with associated ipsilateral distal ulna and radius fractures in an adult patient. Letts et al. found only one out of 33 children who had Monteggia injury in association with fractures of the distal forearm, while Olney and Menelaus reported two out of 102 pediatric patients with a similar injury pattern [4,5]. Monteggia fractures in adults are different to those in children in terms of the mechanism of injury, prognosis, and treatments, and should be considered as a separate entity [3]. We subsequently performed a literature search for a similar injury pattern in adults but this did not return any results.

Monteggia fractures are highly unstable and early recognition of the injury with open anatomical reduction with internal fixation is crucial. However, outcomes of this fracture are still associated with complications, poor functional outcomes and the need for further surgery [6]. There is no established theory for the pathogenesis of Monteggia fracture. Additionally, it is difficult to infer the mechanisms of double fracture, as in this case. We postulate that the most likely mechanism contributing to her injury is a fall on the outstretched hand with a pronated forearm and dorsiflexed wrist, resulting in fractures of the distal radius and ulna [7,8]. The impact of her fall was subsequently transmitted to the elbow, causing a fracture of the radial head and proximal ulna. Her background of osteoporosis and possible prolonged use of corticosteroids (for rheumatoid arthritis and SLE) increased her risk for these injuries and likely led to the extensive multifragmented fractures in her forearm [9]. 

CT scans are increasingly used for the assessment of fractures because they provide more information about the size, pattern, and severity of the fracture as compared to radiographs. Careful analysis of the sagittal and coronal cuts along with 3D reconstruction images can assist the surgeon in determining appropriate surgical exposure and technique, and preparing for appropriate implant selection [9]. In our case, the CT scan showed the extent of the radial head and multifragmented olecranon fracture, which helped us plan and order the equipment required for the surgery. We found that the 3D reconstructions (Figures 3A, 3B) of these complex injuries helped us to understand the extent of the injury and aid preoperative planning and execution of that plan during surgery. This contributed to the good recovery and outcome of our patient, as illustrated by her QuickDASH score of 36.4 which was equivalent to normal (uninjured) age and sex matched controls [10]. Therefore, we could conclude that she had regained normal functional range of movement in her right forearm.

## Conclusions

In conclusion, we report the unusual and rare occurrence of a floating forearm injury: a Monteggia variant injury with an ipsilateral fracture of the distal radius and ulna in an adult patient. This is the first of its case that has been reported in the literature and we have described this complex fracture pattern and fixation technique in detail to provide surgical reproducibility for other surgeons who face this type of injury in the future. This case emphasizes the importance of using appropriate imaging modalities, a multidisciplinary team approach in planning, and dual-consultant operating to obtain good treatment results.

## Appendices

Literature search

PubMed was searched using several search strategies. Exclusion criteria were children, articles published in languages other than English and no full text available. Searching the term “floating forearm” returned 19 results.
